# When Pain Crisis Turns Lethal: A Case Report of Fat Embolism Syndrome in Sickle Cell Disease

**DOI:** 10.3390/jcm15208194

**Published:** 2026-10-21

**Authors:** Abrar J. Alwaheed

**Affiliations:** Department of Internal Medicine, College of Medicine, Imam Abdulrahman Bin Faisal University, King Fahad Hospital of the University, Dammam 31441, Eastern Province, Saudi Arabia; ajwaheed@iau.edu.sa

**Keywords:** sickle cell disease, fat embolism, RBC exchange, plasma exchange, case report

## Abstract

Fat embolism syndrome (FES) is a rare, life-threatening complication of sickle cell disease (SCD), often underrecognized due to overlap with acute chest syndrome, sepsis, and thrombotic microangiopathies. We report a 37-year-old man with sickle cell disease who presented with an acute vaso-occlusive crisis and rapidly deteriorated with respiratory failure, cardiovascular collapse, and neurological dysfunction. Common etiologies including pulmonary embolism, infection, and stroke were excluded. Laboratory evidence of hemolysis, cytopenias, elevated ferritin, and acute cor pulmonale suggested systemic FES secondary to bone marrow necrosis. The patient underwent urgent red cell exchange transfusion, followed by therapeutic plasma exchange and corticosteroids, resulting in significant clinical and hemodynamic recovery. This case highlights the importance of early recognition and aggressive multimodal therapy in SCD-FES.

## 1. Introduction

Sickle cell disease (SCD) is a complex inherited hemoglobinopathy marked by recurrent vaso-occlusion, chronic hemolysis, and progressive end-organ damage. Vaso-occlusive crises (VOCs) represent the most frequent clinical manifestation, affecting approximately 50% of homozygous individuals globally and serving as the primary driver for emergency department visits.

Following VOC, acute chest syndrome (ACS) is the second most common cause of hospitalization, impacting more than half of all pediatric patients during their first decade of life and accounting for up to 25% of all SCD-related mortalities. Within the Kingdom of Saudi Arabia (KSA), where the overall estimated prevalence of SCD is approximately 0.26%, these complications present a substantial national healthcare burden. Local epidemiological data indicate that VOC remains the predominant reason for hospital admission in the KSA, accounting for roughly 55.9% to 64.9% of all SCD-related hospitalizations, while ACS affects approximately 18.1% of hospitalized Saudi SCD patients, positioning it as the leading cause of intensive care unit (ICU) admissions and disease-related mortality nationally.

The baseline prevalence and clinical presentation of acute sickle cell complications vary globally according to specific β-globin gene-cluster haplotypes (Benin, Bantu, Senegal, Cameroon, and Arab–Indian). The Bantu and Benin haplotypes, predominant in sub-Saharan Africa and North America, drive a severe clinical phenotype characterized by early stroke and organ failure. Conversely, the Arab–Indian haplotype, prevalent in India and Eastern Saudi Arabia, often exhibits a milder baseline phenotype due to elevated fetal hemoglobin (HbF) expression. However, this regional genotype presents distinct clinical risks; patients in Saudi Arabia frequently exhibit high baseline hemoglobin and systemic hyperviscosity, paradoxically predisposing them to atypical, catastrophic pulmonary and systemic fat embolism syndrome (FES) during acute crises.

Consequently, FES remains an uncommon but devastating entity that is frequently underdiagnosed. In patients with SCD, FES arises in the absence of trauma and is believed to result from extensive bone marrow necrosis, leading to systemic embolization of fat particles and a profound inflammatory response.

The risk factors predisposing patients to these complications are multifactorial. For standard VOC and secondary ACS, established triggers include deep tissue hypoxia, dehydration, infections, rapid temperature fluctuations, and elevated baseline white blood cell counts. In contrast, the risk profile for FES in SCD is distinct and highly perilous. Key clinical and anatomical risk factors for FES include a history of severe chronic bone destruction and a milder underlying genotype (such as HbSC or HbS*β*+). Crucially, an abnormally high baseline hemoglobin level (>10 g/dL) acts as a major driver; this elevated hemoglobin induces blood hyperviscosity, accelerating sickle-shaped erythrocyte aggregation within the restricted bone marrow vasculature, thereby triggering a latent, multiorgan marrow infarction.

The clinical course of FES in SCD is often fulminant, beginning with an apparently typical painful crisis and rapidly evolving into hypoxemic respiratory failure, neurological deterioration, and multiorgan dysfunction. Diagnostic uncertainty is common due to significant overlap with other acute SCD complications, and delays in recognition are associated with high mortality. Management strategies rely heavily on prompt recognition, though the existing standard of care varies significantly between general FES and FES occurring in the context of SCD. In non-SCD populations (such as orthopedic trauma patients), the standard of care for FES remains purely supportive. However, in sickle cell disease, standard supportive monitoring is often insufficient due to the ongoing hemolytic and vaso-occlusive cascades. Prevention of FES in high-risk SCD patients during an acute VOC relies on aggressive hydration, optimal multimodal pain management, and incentive spirometry. Once FES is suspected or established in an SCD patient, the definitive therapeutic standard of care shifts from conservative monitoring to immediate, aggressive intervention. Emerging evidence, however, suggests that early intervention, particularly red cell exchange transfusion, with or without therapeutic plasma exchange can be lifesaving.

We present a striking case of systemic fat embolism syndrome in a patient with sickle cell/β^0^-thalassemia who experienced sudden cardiopulmonary collapse and neurological impairment. This report aims to heighten clinical awareness of FES in SCD, emphasize key diagnostic features, and illustrate the potential for favorable outcomes with timely, aggressive management.

## 2. Case Presentation

A 37-year-old man with sickle cell–β^0^-thalassemia and G6PD deficiency was admitted with acute vaso-occlusive and hemolytic crises. Laboratory evaluation revealed a hemoglobin level of 10.9 g/dL. Lactate dehydrogenase (LDH) and platelet counts remained stable at the patient’s baseline (300–333 U/L and 144–163 × 10^9^/L, respectively), matching values documented two months prior. No earlier laboratory records were available. The patient’s baseline reticulocyte count historically ranged between 3.0% and 4.2%. His medical history was notable for recurrent acute chest syndrome requiring multiple ICU admissions at an external facility. His home medications included daily folic acid (5 mg) and hydroxyurea (1500 mg). Chronic complications of his disease included avascular necrosis of the left shoulder and bilateral femoral heads (status post right total hip arthroplasty), vertebral compression fractures, and chronic non-bacterial osteomyelitis (CNO). The CNO was diagnosed in 2019 at an outside institution and characterized by recurrent episodes that required multiple incision and drainage procedures, all of which yielded sterile cultures. He was managed by antibiotics and maintained on suppressive therapy with doxycycline 100 mg orally twice daily, 10 mg oral prednisolone tapering dose, and methotrexate 20 mg once weekly.

On admission, he was afebrile, BP 125/60 mmHg, HR 86 bpm, RR 18/min, and O_2_ saturation 100% on room air. He was managed with supportive care including 0.45% normal saline intravenous fluid, incentive spirometry and opioid analgesics. Within hours, he developed severe chest pain and respiratory distress, tachycardia (HR 136 bpm), and required 5 L/min supplemental oxygen via nasal cannula. Chest X-ray and CT pulmonary angiography (CTPA) excluded pneumonia and pulmonary embolism. Labs revealed worsening hemolysis, with a drop of hemoglobin to 7.7 gm/dL (Table 1) necessitating the temporary discontinuation of hydroxyurea and methotrexate therapies.

He became vitally unstable, and required intubation for suspected ACS. He experienced pulseless electrical activity (PEA) cardiac arrest and revived after six minutes. He subsequently developed supraventricular tachycardia and atrial fibrillation, managed with electrical cardioversion. Transthoracic echocardiography (TTE) revealed mildly reduced ejection fraction (EF) of 40%, septal motion abnormalities consistent with right ventricle (RV) volume and pressure overload, moderately dilated RV, moderate-to-severe systolic dysfunction, elevated right ventricle systolic pressure (RVSP) of 60 mmHg, and moderate-to-severe tricuspid regurgitation.

He was admitted to the ICU, required vasopressors, and underwent urgent RBC exchange, reducing HbS from 73% to 11.7%. Electroencephalography (EEG) showed continuous bilateral theta/delta activity, consistent with diffuse cerebral dysfunction, without epileptiform discharges.

FES was suspected after exclusion of other causes. Subsequently, therapeutic plasma exchange (TPE) was initiated for seven days, alongside corticosteroids. Peripheral smear revealed neutrophilic leukocytosis with toxic granulation and few reactive lymphocytes; ferritin was elevated (4481 ng/mL), and initial septic workup was negative. Supportive care was initiated including antibiotics for hospital-acquired pneumonia and PRBC transfusions. Brain MRI revealed subacute lacunar infarction in the right centrum semiovale, diffuse microhemorrhages, small vessel ischemic changes, and focal cortical enhancement in the right posterior parietal region (Figure 1).

Follow-up TTE demonstrated normalized EF (55%) and RVSP (31 mmHg). The patient showed remarkable clinical improvement without surgical interventions, was started on an extensive rehabilitation program, and discharged on his previous hydroxyurea regimen after hematological and clinical recovery.

## 3. Discussion

Sickle cell disease is a hereditary hemoglobinopathy caused by a single nucleotide substitution in the β-globin gene, resulting in the production of abnormal hemoglobin S (HbS). The disease is characterized by recurrent vaso-occlusive crises and multisystem complications [1].

In this paper we present fat embolism syndrome in a patient with sickle cell disease, which is an uncommon and frequently underrecognized complication, yet it is associated with substantial morbidity and mortality.

Initial evaluation of our patient revealed critical hematological red flags signaling impending marrow failure and systemic collapse. Despite active hemolysis, the patient presented with a paradoxically high hemoglobin level. This was compounded by a borderline low platelet count, indicating early peripheral consumption or marrow suppression. Crucially, an inappropriately low reticulocyte count was observed, confirming acute bone marrow aplasia likely driven by the synergistic myelosuppressive effects of his chronic medications under acute stress. Profound systemic inflammation was highlighted by a markedly elevated serum ferritin [2].

FES most commonly occurs in the setting of trauma and orthopedic interventions, particularly following long-bone fractures with an incidence ranging from 1% to 29%, reflecting substantial variability in diagnostic criteria. In contrast, individuals with SCD are at risk for non-trauma-related FES, which arises independently of mechanical injury. However, FES in sickle cell disease remains an infrequent but serious complication, accounting for 10.3% in one metanalysis [3]. Evidence suggests that subclinical marrow necrosis and chronic low-grade fat embolization may occur even during the steady state in SCD, supported by observations of persistently elevated secretory phospholipase A_2_ (sPLA_2_) levels in affected individuals [4]. FES in sickle cell disease has historically been associated with substantial mortality, reported to be as high as 64%. It can result in multiorgan dysfunction, most notably type 1 respiratory failure, hypoxemia, tachypnea, acute respiratory distress syndrome (ARDS), and acute cor pulmonale.

Analysis of published cases demonstrates a temporal decline in mortality, from 66% in reports prior to 2013 to 33% between 2014 and 2018, largely attributable to the timely implementation of red cell exchange (RCE) therapy [4].

A comprehensive literature review published through 2018 identified 87 cases and demonstrated that FES disproportionately affects milder sickle cell genotypes. Hemoglobin SC disease (HbSC) was the most frequently represented variant, accounting for approximately 50% of reported cases, whereas sickle/β^+^-thalassemia (HbSβ^+^) represented an additional 18% [4], which is the case in our patient. By contrast, homozygous sickle cell disease constituted only about 15% of cases [4]. One prevailing hypothesis posits that individuals with non-SS genotypes exhibit higher baseline hemoglobin and hematocrit levels, increasing blood viscosity and susceptibility to bone marrow ischemia and necrosis. However, emerging data complicate this paradigm; a recent single-center cohort reported that most FES cases (70%) occurred in patients with the HbSS genotype, suggesting that alternative mechanisms contribute to FES risk in this population [5]. This trend may also reflect regional demographic variations, as the prevalence of the HbSS genotype is higher in certain countries, mirroring our patient’s background.

Infections such as human parvovirus B19 and COVID-19 are recognized precipitating factors for fat embolism syndrome in sickle cell disease through immune-mediated mechanisms and heightened systemic inflammation [6,7,8]. However, an infectious etiology was successfully ruled out in the reported patient following a negative septic workup.

The diagnosis of fat embolism syndrome, particularly in the context of SCD, remains primarily clinical. The absence of standardized and specific diagnostic criteria for non-traumatic FES in SCD significantly complicates recognition and timely diagnosis. Traditional diagnostic frameworks developed for traumatic FES, such as the Gurd and Wilson criteria and the Schonfeld scoring system, lack specificity in this population and may overlap with other acute SCD complications [4,5,9,10]. All these differential diagnoses in addition to pulmonary embolism were excluded in our patient. The patient strongly satisfied both scoring paradigms on acute deterioration, as described in Table 2.

The classic presentation of FES is a triad of respiratory failure, neurologic complications, and petechial rash, which do not necessarily present in all patients with suspected diagnosis [11]. A high index of clinical suspicion is therefore critical, particularly in patients with sickle cell crisis who develop acute hypoxemia, fever, and altered mental status. FES often presents initially as a painful vaso-occlusive crisis, followed by the development of type 1 respiratory failure and neurological manifestations, typically within 48–72 h.

FES can closely mimic thrombotic thrombocytopenic purpura (TTP), which may result in diagnostic confusion and mismanagement [12,13]. This possibility was raised and excluded during the initial evaluation and deterioration of our patient. Key distinguishing features include the pattern of organ involvement and hematological findings. Respiratory failure is uncommon in TTP, whereas it is a hallmark of FES. Additionally, TTP typically presents with more pronounced thrombocytopenia and abundant schistocytes on peripheral blood smear (PBS). In contrast, FES often demonstrates a leukoerythroblastic picture with only occasional schistocytes on PBS. Careful clinical and laboratory assessment in addition to liaison with a hematopathologist is crucial [13].

Cardiac ultrasonography can support the diagnosis of FES by demonstrating fat particles in the right heart chambers or signs of acute cor pulmonale, provided that pulmonary thromboembolism is excluded. In the present case, a chest CT scan and a transthoracic echocardiogram were performed. Following a multidisciplinary evaluation by radiology and cardiology specialists, acute pulmonary embolism was ruled out. However, the TTE revealed acute heart failure with reduced ejection fraction (HFrEF), which is consistent with cardiovascular manifestations of FES [5].

The pathogenesis of fat embolism syndrome in both traumatic and non-traumatic conditions, including SCD, involves a dual mechanism of mechanical vascular obstruction and biochemical inflammatory injury. From a mechanical perspective, fat globules liberated during traumatic injury or extensive bone marrow necrosis enter the venous circulation and lodge within the pulmonary microvasculature. This physical obstruction leads to a profound ventilation–perfusion mismatch, which can ultimately precipitate ARDS [11].

Conversely, the biochemical theory suggests that circulating free fatty acids and lipolytic degradation products exert direct cytotoxicity on endothelial cells and pneumocytes. These metabolites trigger an inflammatory cascade driven by complement activation, cytokine release, and neutrophil recruitment that increases vascular permeability, drives diffuse alveolar damage, and promotes multiorgan dysfunction. In sickle cell disease, this response is further amplified by baseline hemolysis, oxidative stress, and endothelial activation, increasing susceptibility to severe FES. Notably, the time required for this toxic metabolic accumulation accounts for the characteristic 24–48 h clinical delay observed in the present case [11].

Neurological manifestations are common, ranging from mild confusion and headache to coma, seizures, rigidity, and focal deficits, with severity increased in the presence of a right-to-left intracardiac shunt (e.g., PFO) [4].

Brain MRI may demonstrate the characteristic “starfield pattern”, defined by punctate foci of diffusion restriction with associated microhemorrhages in the white matter. However, a systematic review by Shadmani et al. reported that the starfield pattern of restricted diffusion is present in 65.4% of cases during the acute phase and is less frequently observed beyond five days after symptom onset [8,14]. This may explain the absence of typical MRI findings in our patient, in addition to the inability to perform magnetic resonance angiography [6].

A recent single-center cohort study by Salam A. et al. reported a further reduction in mortality to 18.5% [5]. Fatal outcomes are most commonly observed in younger adults presenting with profound cardiorespiratory compromise and severe hepatic dysfunction. Among survivors, severe neurological deficits occur in approximately 14% [7], although a significant proportion exhibit substantial neurological recovery with rehabilitation. One cohort reported complete or near-complete long-term recovery in 63% of patients, highlighting the potential for favorable outcomes with early recognition and intervention [4].

Other reported features include petechial rash (often in the upper trunk, head, or neck), which can present in less than 50% of cases; retinal changes such as perivascular whitening or cotton wool spots; and rarely blindness [11].

The management of FES in sickle cell disease integrates prompt supportive care with targeted interventions to halt the sickling cascade and mitigate systemic inflammation. Initial therapy focuses on maintaining adequate oxygenation, hemodynamic stability, and organ perfusion. Consequently, respiratory support ranging from supplemental oxygen to mechanical ventilation is indicated for patients with hypoxemia or ARDS. Concurrently, aggressive hydration is employed to optimize microvascular flow and prevent further microvascular sickling.

Early initiation of red cell exchange is lifesaving and remains the mainstay of therapy once FES is suspected [4]. The primary goal is to reduce hemoglobin S levels to below 10–20%, interrupting the sickling cycle and preventing further bone marrow necrosis. RCE also facilitates the removal of leukocytes, cytokines, fat globules, and platelets, thereby attenuating inflammation and hyperviscosity. Evidence consistently demonstrates that RCE improves survival compared with simple transfusion or no transfusion. RCE was initiated in our patient within 24 h of clinical deterioration with final HbS of 11.7% [8]. Although the patient carries risk factors for FES, his stable clinical status, despite mild thrombocytopenia, does not fully justify pre-emptive red cell exchange. Given the procedural risks of RCE, intensive monitoring and supportive care remain the most prudent approach, reserving emergency exchange for active clinical decompensation.

Therapeutic plasma exchange (TPE) is increasingly recommended as an adjunct to RCE in severe or refractory cases. By removing circulating fat droplets and pro-inflammatory cytokines from bone marrow necrosis, TPE can accelerate recovery and improve outcomes [5,8]. This strategy was followed in our patient, with daily TPE guided by his clinical and laboratory response.

Case reports and series have documented the successful use of TPE in SCD-FES, often resulting in complete recovery. The duration of treatment can be determined by clinical response, with serial monitoring of ferritin, lactate dehydrogenase, and platelet counts [8,13,15]. Optimal outcomes require early involvement of critical care, hematology, and neurology teams. Investigational therapies, including targeted anti-inflammatory agents, may provide additional benefit in severe or refractory cases.

Corticosteroids, although primarily used in traumatic FES, have anti-inflammatory effects that may benefit SCD-FES. They also inhibit complement-mediated leukocyte aggregation, stabilize cell membranes, and reduce circulating free fatty acids. Meta-analytic data suggest that steroids may decrease FES risk by up to 77% in long-bone fracture patients.

Optimal outcomes require early involvement of critical care, hematology, and neurology teams. Investigational therapies, including targeted anti-inflammatory agents, may provide additional benefit in severe or refractory cases.

## 4. Conclusions

Fat embolism syndrome in sickle cell disease is a rapidly progressive, life-threatening complication, even in seemingly mild cases. Survival hinges on swiftly recognizing sudden systemic collapse with acute hypoxemia, neurological deterioration, and cytopenias. Red cell exchange is lifesaving, halting the sickling cascade and limiting marrow necrosis, while therapeutic plasma exchange can remove circulating fat and inflammatory mediators in severe cases. Combined with supportive care and early rehabilitation, rapid intervention is key to preventing multiorgan failure and improving long-term outcomes.

## Figures and Tables

**Figure 1:**
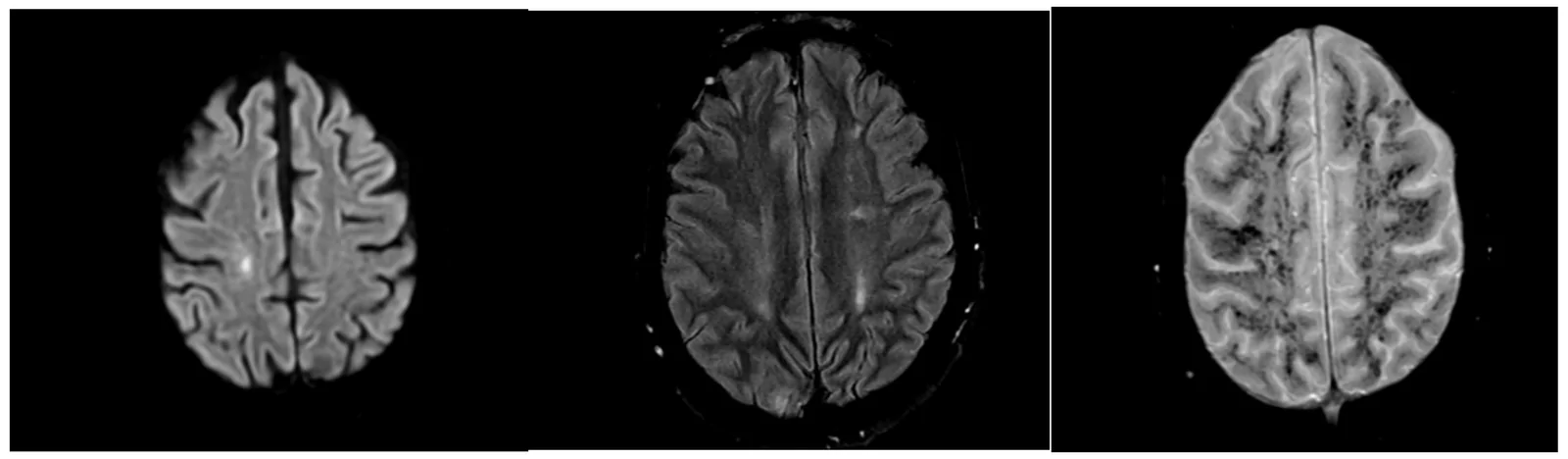
Brain MRI (FLAIR, DWI and SWI) showing several hyper-intense lesions in both hemispheres indicating old ischemic changes and multiple susceptible areas indicating microhemorrhages, raising the possibility of fat embolism syndrome.

**Table 1:** Laboratory investigations from admission until discharge.

Days	WBC (4.0–11.0) (10^3^/μL)	Hgb (13.0–18.0) (gm/dL)	Hct. (42.0–52.0)%	RBC (4.70–6.10) 10^6^/μL	Platelet (140.0–450.0) (10^3^/μL)	Reticulocyte (1.0–3.0) (%)	T. Bilirubin (0.3–1.2) (mg/dL)	D. Bilirubin (0.10–0.50) (mg/dL)	LDH (125–220) (U/L)
1	6.1	10.9	35.7	3.98	140	1.1	2.3	0.8	321
2	12.7	7.7	22.9	3	84	1.3	7.9	0.63	2838
7	3.7	7.5	23.5	2.68	36	1.4	1.3	0.47	1747
10	4.1	8.1	25.1	2.80	58	1.9	1.7	0.69	622
Discharge	5.9	8.5	28.3	3.26	238	3.3	1	0.44	292

WBC: white blood cell count, Hgb: hemoglobin, Hct: hematocrit, RBC: red blood cell, T. bilirubin: total bilirubin, D. bilirubin: direct bilirubin, LDH: lactate dehydrogenase.

**Table 2:** Diagnostic criteria and patient scoring for fat embolism syndrome (FES).

Diagnostic Framework Required Threshold	Patient Presentation and Findings		Criteria Status
**Gurd and Wilson Criteria** (≥2 Major criteria) OR (1 Major + 4 Minor criteria)	**Major Criteria Met**	**Findings**	2 Major and 3 Minor criteria
	Respiratory insufficiency	Severe distress, emergent intubation	
	Neurological involvement	Diffuse cerebral dysfunction on EEG; subacute lacunar infarct on MRI	

	**Minor Criteria Met**	**Findings**	
	Tachycardia	136 bpm	
	Sudden anemia	Hemoglobin acute drop to 7.7 gm/dL	
	Thrombocytopenia	Borderline low platelet counts on admission	
**Schonfeld Fat Embolism Index** Cumulative score of ≥ 5 points	Hypoxemia/severe respiratory failure (3 points) Tachycardia >120 bpm (1 point) Tachypnea/respiratory distress (1 point) Confusion/neurological depression (1 point)		Total Score: 6 points
**FES-SCD Scoring System** **(Sickle Cell Disease Specific)** Cumulative score of ≥ 3 points	Neurological symptoms (2 points Chest pain/acute chest syndrome features (1 point) Sudden anemia/rapid drop in hemoglobin (1 point) Thrombocytopenia/borderline low platelets (1 point)		Total Score: 5 points

## Data Availability

The data that support the findings of this study are not openly available due to reasons of sensitivity and are available from the corresponding author upon reasonable request.

## Abbreviations

Abbreviation	Meaning
ACS	Acute Chest Syndrome
ALP	Alkaline Phosphatase
ARDS	Acute Respiratory Distress Syndrome
AVN	Avascular Necrosis
BMN	Bone Marrow Necrosis
BP	Blood Pressure
Bpm	Beats per minute
CNO	Chronic Non-bacterial Osteomyelitis
CNS	Central Nervous System
COVID-19	Coronavirus Disease 2019
CT/CTPA	Computed Tomography/Computed Tomography Pulmonary Angiography
D. bilirubin	Direct Bilirubin
DWI	Diffusion-Weighted Imaging
EEG	Electroencephalography
EF	Ejection Fraction
ESR	Erythrocyte Sedimentation Rate
FES	Fat Embolism Syndrome
FES-SCD	Fat Embolism Syndrome–Sickle Cell Disease-specific index
FFAs	Free Fatty Acids
FLAIR	Fluid-Attenuated Inversion Recovery
G6PD	Glucose-6-Phosphate Dehydrogenase
Hct	Hematocrit
Hgb/Hb	Hemoglobin
HbS	Hemoglobin S (Sickle hemoglobin)
HbSS/HbSC	Homozygous Sickle Cell Genotype/Hemoglobin SC Variant Genotype
HPV B19	Human Parvovirus B19
HR	Heart Rate
HFrEF	Heart Failure with reduced Ejection Fraction
ICU	Intensive Care Unit
KSA	Kingdom of Saudi Arabia
LDH	Lactate Dehydrogenase
MRI	Magnetic Resonance Imaging
O_2_	Oxygen
PaO_2_	Partial pressure of Oxygen in arterial blood
PBS	Peripheral Blood Smear
PEA	Pulseless Electrical Activity
PFO	Patent Foramen Ovale
PRBC	Packed Red Blood Cell
RBC/RCE	Red Blood Cell count/Red Cell Exchange
RR	Respiratory Rate
RV	Right Ventricle
RVSP	Right Ventricle Systolic Pressure
SARS-CoV-2	Severe Acute Respiratory Syndrome Coronavirus 2
SCD	Sickle Cell Disease
sPLA_2_	Secretory Phospholipase A_2_
SWI	Susceptibility-Weighted Imaging
T. bilirubin	Total Bilirubin
TPE	Therapeutic Plasma Exchange
TTP	Thrombotic Thrombocytopenic Purpura
TTE	Transthoracic Echocardiography
VOC	Vaso-Occlusive Crisis
WBC	White Blood Cell count
